# Serum profile of cytokines and their genetic variants in metabolic syndrome and healthy subjects: a comparative study

**DOI:** 10.1042/BSR20181202

**Published:** 2019-02-01

**Authors:** Uzma Zafar, Saba Khaliq, Hafiz Usman Ahmad, Khalid Pervaiz Lone

**Affiliations:** 1 Department of Physiology and Cell Biology, University of Health Sciences, Lahore, Pakistan; 2 Department of Physiology, Lahore Medical and Dental College, Lahore, Pakistan

**Keywords:** IL-6, insulin resistance, Metabolic syndrome, TNF-α

## Abstract

Aim: To identify genetic variants in promoter areas of IL-6 -174 G>C and TNF-α -308 G>A in metabolic syndrome (Met S) and controls and associate them with Met S and serum cytokine levels.

It was a cross-sectional study, including 224 cases of Met S and 200 controls. A fasting blood sample was taken and biochemical parameters including serum glucose, insulin, lipid profile, interleukin-6 (IL-6) and tumor necrosis factor α (TNF-α) were measured. Restriction fragment length polymorphism was used to identify the genetic variants of IL-6 and TNF-α. Serum levels of IL-6 and TNF-α and insulin resistance were significantly higher in cases than the controls. IL-6 showed significant positive correlation with HOMA-IR and TNF-α. CC genotype of IL-6 was associated with the increased risk of Met S (*P*=0.016, OR for CC vs GC+GG = 2.33, CI: 1.15–4.71). There was no significant difference of TNF-α genotypes between the cases and the controls. Serum TNF-α and IL-6 levels were significantly higher in AA and CC genotypes of TNF-α (-308 G>A) and IL-6 (-174 G>C) as compared with the GG (*P*=0.00 and *P*=0.001). Significant correlation of IL-6 with TNF-α and insulin resistance was observed that may provide us a therapeutic target for preventing metabolic derangements from insulin resistance.

## Introduction

Cytokines are pleiotropic multifaceted polypeptides having versatile actions. The robust role of these molecules has been implicated in metabolic syndrome (Met S) and related traits. Met S also named as insulin resistance syndrome (IRS) is a clustering of clinical and biochemical abnormalities such as central obesity, hypertension, impaired blood lipid and glycemic parameters. All these derangements lead to type 2 diabetes mellitus (T2DM), acute coronary syndrome (ACS), cerebrovascular accident, non-alcoholic steatohepatitis (NASH) and renal failure [1,2]. Excess body fat deposition especially perivisceral and omental adipose tissue results in an atmosphere of chronic low grade inflammation. These areas are infiltrated with macrophages, lymphocytes and myeloid series suppressor cells. Vicious cycle of activation of innate immunity starts resulting in the release of cytokines like interleukin-6 (IL-6) and tumor necrosis factor α (TNF-α); these peptides establish the link between central adiposity, inflammation, insulin resistance (IR) and susceptibility to its various complications [3]. The cytokines produced by the obese adipose tissue result in the recruitment of more monocytes in the adipose organs and accelerate their differentiation into M1 macrophages. These activated macrophages produce more cytokines driving more T lymphocyte migration further cytokine production and progression of chronic inflammation associated with metabolic diseases [4]. TNF-α generates IR by various mechanisms including serine phosphorylation of insulin receptor substrate-1 (IRS-1), altered lipid metabolism in adipocytes with increased release of free fatty acids in blood, impaired differentiation of pre-adipocytes to adipocytes by inhibiting PPPAR-γ (peroxisome proliferator activated receptor γ) genes and CEBP (CCAAT enhancer binding protein) resulting in the expansion of adipose mass. The inflammatory cytokine TNF-α released by the perivisceral adipose tissue also enhances the activation of the transcription factor nuclear factor κB (NFκB), which worsens metabolic derangements by up-regulating the activity of the cytokine genes in the peripheral tissues and increased oxidative stress [5,6]. IL-6, another multiaction cytokine, is released from adipocytes and macrophages of white adipose tissue and also from skeletal muscles and liver. Action of IL-6 varies depending upon the type of the target tissues and metabolic state. In muscles, at the time of exercise, it has anti-inflammatory and insulin sensitizing action but in adipocytes and liver it generates IR by SOCS3 (suppressor of cytokine 3 signaling) pathway that impairs IRS-1 phosphorylation [7].

In the context of Met S, cytokine genetics especially the single nucleotide variants of the regulatory areas determine the progression of inflammation and response to therapy by affecting the levels of cytokines in the blood. Genetic variability among different populations also helps us in understanding the diversity of the disease course and susceptibility to its complications [8]. Certain studies have provided evidence for the association of the single nucleotide promoter polymorphism of TNF-α and IL-6 with the severity of the disease process in Met S while others are non-conclusive [9–11]. A systematic meta-analysis by Hua et al. [12] reported significant association of TNF-α -308 promoter polymorphism with the stages of myocardial infarction in Caucasians and Asians. However, no significant association of TNF-α promoter polymorphism with coronary artery disease and myocardial infarction was found in a case–control study on the Han Chinese population [13]. Another study on Brazilians reported association of promoter polymorphisms of IL-6 and TNF-α with changes in lipid and glucose metabolism induced by the life style intervention [14]. Due to ethnic differences, results of the different studies are variable and equivocal. In the previous two studies in Pakistan, two different results were reported. In a study by Satti et al. [15], CC genotype and C allele of -174 C>G IL-6 promoter polymorphism was found to be associated with coronary artery disease. No CC genotype was found in the controls. In the other study, genotype GG of -174 G>C IL-6 was found to have significant association with T2DM and increased serum IL-6 levels in Rajput ethnic group [16]. Present study was designed with the objectives to determine genetic variability in the promoter areas of TNF-α (-308 G>A rs1800629) and IL-6 (-174 G>C rs1800795) in Met S and healthy subjects and associate the SNPs with Met S and cytokine levels in the blood. Identification of these cytokines as modulator of metabolic derangements leading to the end organ damage may provide us new therapeutic targets. Treatment regimens can be altered or eased out for T2DM and its various complications.

## Subject and methods

### Ethical considerations

The study was approved by the Institutional Review Board of University of Health Sciences, Lahore. All participants were fully informed of the study and written informed consent was taken.

### Study subjects

This was a cross-sectional comparative study, conducted in the Department of Physiology and Cell Biology, University of Health Sciences, Lahore. Study population included 224 cases of Met S and 200 healthy controls. Sample size was calculated by following the equation ‘Hypothesis test for two population proportions’ from WHO calculator. Level of significance was 0.05 and power of the study (1-β) was taken as 90% [17]. The data were collected from Sheikh Zayed Hospital, Lahore over the time span of 6 months July to December 2016. Recruited cases of Met S were outpatients who had been registered in the Diabetic Clinic of the Sheikh Zayed Hospital. They were approached retrospectively in the Diabetic clinic. The diabetic clinic was working 6 days a week and 50–60 registered patients visited daily. A questionnaire was used as a study tool and data were collected on demographics, disease history, medications, physical and biochemical parameters. Met S was defined according to the International Diabetes Federation criteria [18]. All selected subjects of Met S were centrally obese, i.e., males were having waist circumference of ≥90 cm and females ≥80 cm with any two of the following four features: (1) serum triglycerides (TGS) ≥150 mg/dl or on treatment for TGs, (2) serum high density lipoprotein cholesterol (HDL-c) <40 mg/dl in men and 50 mg/dl in women or on treatment for dyslipidemias, (3) blood pressure (BP) >130/85 or on treatment for hypertension and (4) fasting serum glucose >100 mg/dl or on treatment for DM. If any two of the four traits were present along with central obesity subjects were diagnosed to have Met S. Available data and investigations with the subjects were checked. Patients already taking anti-hypertensives and lipid lowering drugs like statins, niacin or fibric acid derivatives were considered to be hypertensive and dyslipidemic, irrespective of their current blood pressure and lipid profile [2,18]. After establishing the diagnosis of Met S, fasting status of the subjects was inquired and fasting was defined as no caloric intake for the last 8–10 h [19]. Finally, those cases of Met S having an overnight fast of 8–10 h were included on the particular visit. All those having evidence of acute infection, end-stage renal disease, hepatic decompensation, chronic infective and inflammatory states or secondary causes of diabetes mellitus were excluded. In Met S group 79% were diabetics, 9% were having impaired fasting glucose, 78% gave the history of hypertension and were on anti-hypertensives, 93% were having dyslipidemias and on lipid-lowering agents. Age- and sex-matched controls were selected from the general population. Selection criteria for the controls was waist circumference ≤90 cm in men and ≤80 cm in females, non-hypertensives and non-diabetics. There was no history of intake of lipid-lowering agents or any medications for diabetes and hypertension. Fasting and 2-h postprandial blood sugar and blood pressure of controls were checked on two separate days. Those subjects were included having fasting blood sugar <100 mg/dl, 2 h after meals <140 mg/dl and BP <130/85 [2,18]. All those having evidence or history of metabolic, chronic infective or inflammatory states were excluded.

### Clinical and biochemical measurements

Waist circumference and blood pressure were recorded by the standard methods. Waist circumference was measured at the mid-point between the lower border of the last rib and the upper rim of the antero-superior iliac crest. BP was recorded from the left arm in sitting position after 10 min of rest. Two measurements were taken after 5-min interval and an average of two recordings was noted (Ferreira-Hermosillo et al., 2015). Fasting blood sample of 8 ml was taken from the subjects having overnight fast of 8 to 10 h to study different parameters. Blood for DNA extraction was stored at −20°C, and serum was separated and stored at −80°C. Fasting serum glucose and lipid profile including serum TGs, HDL-c and cholesterol were measured by the colorimetric method (Randox Kits, U.K.). Insulin was measured by Human insulin ELISA kit (Elabscience, Germany). Insulin resistance was calculated from fasting serum glucose (mmol/l) and fasting serum insulin (µIU/ml) by HOMA-IR (homeostatic model assessment for insulin resistance) using following formula [20]:

HOMA-IR=Fasting serum glucose×Fasting serum insulin/22.5

Serum levels of TNF-α and IL-6 were measured by ELISA using TNF-α and IL-6 ELISA kits (Elabscience, Germany).

### Analysis of genetic variants

DNA was extracted from FavorPrep Blood Genomic DNA Extraction Kit (Taiwan, China) according to the manufacturer’s guidelines. DNA yield was checked by nanodrop followed by gel electrophoresis. IL-6 -174 G>C PCR was carried out in 25 µl reaction mixture containing 2 µl (50 ng/µl) of DNA, 2XPCR master mixture (Fermetas, U.S.A.) and 0.56 µM Forward 5′-ATGACTTCAGCTTTACTCTT-3`and Reverse 5′-ATAAATCTTTGTTGGAGGGT3 `primers (Macrogen, Inc. Korea).The PCR conditions used for the amplification of IL-6 gene region −174G/C SNP were as follows: initial denaturation at 95°C for 4 min followed by 35 cycles of denaturation at 95°C for 45 s, annealing at 60°C for 30 s, and extension at 72°C for 60 s followed by a single final extension step at 72°C for 10 min. Restriction fragment length polymorphism (RFLP) was carried out in a 20 µl reaction volume containing 10 µl PCR product and 5 U of Nla III restriction enzyme (Thermos Fisher Scientific; U.S.A.) and incubated at 37°C overnight. The results of RFLP were as follows: GG wild-type genotype yield two fragments of 216- and 28-bp, CC genotype yield three fragments of 122-, 94- and 28-bp, and GC genotype yield four fragments of 216-,122-, 94- and 28-bp [21]. TNF-α -308G>A PCR was carried out in 25 µl reaction mixture, using Forward 5′-AGGCAATAGGTTTTGAGGGCCAT-3′ and Reverse 5′-GCCTCAGAGACATCTCCAGTC-3′ primers to amplify 107-bp fragment. The amplified product was subjected to restriction digestion by Nco1 restriction enzyme (Thermos Fisher Scientific, U.S.A.) and incubated at 37°C overnight. Restricted fragments were run on 3% agarose gel and analyzed as GG fragment 87- and 20-bp, AA fragment 107-bp, GA fragment 87-, 20-and 107-bp [22].

In order to know the inflammation rate mRNA expression of IL-6, TNF-α and NFκB were determined after extracting RNA from freshly collected blood samples (10 samples of cases and controls each). The RNA was reverse transcribed using reverse transcriptase PCR and cDNA was synthesized with the Fermentas first stand cDNA synthesis kit (Fermentas, U.S.A.). Real Time PCR of TNF-α, IL-6 and NFκB was done to determine the mRNA levels. PCR primers for: TNF-α- F-′GGAGAAGGGTGACCGACTCA′ and R-′CTGCCCAGACTCGGCAA′-; for NFκB-F-′TACTCTGGCGCAGAAATTAGGTC′ and R- ′CTGTCTCGGAGCTCGTCTATTTG′ for IL-6- F-′AGCCCTGAGAAAGGAGACATGTA′ and R-′TCTGCCAGTGCCTCTTTGC′; and β actin-F-′TCCACCTTCCAGCAGATGTG′ and R-′GCATTTGCGGTGGACGAT′. The expression was determined by mixing the reagents including 2× Syber Green real time master mix, template RNA, primers and RNAase free water according to the manufacturer’s guidelines. About 0.5 µl of RNA was added to the PCR plates and program of the machine was set according to the manufacturer’s instructions [23].

## Statistical analysis

The data were entered and analyzed using SPSS version 22.0 (Statistical Package for Social Sciences). Normal distribution of the data was checked by Shapiro–Wilk’s statistics. Mean ± SD (Standard deviation) were given for normally distributed quantitative variables and median with IQR (interquartile range) for non-normally distributed quantitative variables. Student’s *t-*test and Mann–Whitney *U-*test were applied to compare normally and non-normally distributed quantitative variables between the cases and the controls. Spearman correlation was applied to see the relation between the cytokines levels and other study parameters. Student’s *t-*test was applied to see the difference between the mRNA expression of IL-6, TNF-α and NFκB between the cases and the controls.

Genotype and allele frequency followed by Hardy–Weinberg equilibrium was calculated by online genetic epidemiology tool (OEGE) (http//www.oege.org) using the Pearson goodness of fit *X^2^* test with one degree of freedom for bi-allelic markers. In order to study the frequency and association of polymorphisms with the study groups, three genetic models (Co-dominant Model, Dominant Model and Recessive Model) were constructed. Genotype frequencies of two groups were compared by chi-square test (χ^2^) and odds ratio was calculated. Logistic regression was applied to observe the association between SNP and Met S after controlling for the possible confounders such as age and sex.

## Results

### Characteristics of the study population

Out of total 424 subjects, 224 were cases of Met S and 200 healthy controls. Of these 77% were males and 23% females in both groups. The mean ± SD of ages of subjects with Met S was 47.03 ± 8.03 years and of controls was 46.54 ± 8.19 years. There was no significant difference in mean ages of two groups. Duration of Met S was of less than a year in 60% of the cases and more than a year in 40% of the cases. Mean duration of Met S was 3.92 ± 4.25 years. Subjects with Met S had significantly higher waist circumference, BP, serum triglyceride, glucose and insulin levels compared with the controls (*P*=0.00). Insulin resistance measured by HOMA-IR was also significantly higher in the cases than the controls (*P*=0.00). Serum TNF-α and IL-6 levels were significantly higher in the cases than the controls (*P*=0.001). Comparison of biochemical parameters is given in Tables 1 and 2.

**Table 1:** Comparison of clinical and biochemical parameters of the study groups

Clinical and biochemical parameters	Metabolic syndrome (*n*=224) (178/46)	Healthy group (*n*=200) (165/35)	*P* value
Age in years	47.03 ± 8.03	46.54 ± 8.19	0.491
Systolic BP in mm of mercury	125.82 ± 15.40	114.64 ± 15.00	<**0.0001^†^**
Diastolic BP in mm of mercury	80.43 ± 10.5	75.53 ± 8.7	<**0.0001^†^**
Waist circumference in centimeters	101.30 ± 8.87	80.56 ± 10	<**0.0001^†^**
Body mass index	28.85 ± 5.19	23.34 ± 3.12	<0.001^†^
HDL (mg/dl)	39.63 ± 9.20	39.99 ± 4.92	0.553
Triglycerides (mg/dl)	187 (140–270)	150.75 (131–177)	<0.0001*
Cholesterol (mg/dl)	209 (180–243)	178 (158–-191)	<0.0001*
Serum uric acid (mg/dl)	6.63 ± 1.52	5.34 ± 1.05	<0.0001^†^
Fasting glucose (mmol/l)	8.8 (6.6–10.9)	4.9 (4.4–5.2)	<0.0001*
Fasting insulin (µg/ml)	27.7 (16–48)	8.5 (5.8–11)	<0.0001*
HOMA-IR	10.4 (5.7–18)	1.9 (1.2–2.5)	<0.0001*
TNF-α (pico g/ml)	20.5 (10–62)	9.5 (6.1–18)	<0.0001*
IL-6 (pico g/ml)	16 (7–46)	7.5 (4.3–20)	0.001*

Values given are Mean ± SD or Median (IQR) as related to the results obtained through the Shapiro–Wilk’s statistics. Bold ‘*P*’ values are generated by Student’s *t-*test^†^ and Mann–Whitney *U*-test*. A *P* of <0.05 is statistically significant.

HOMA-IR = Fasting serum glucose × Fasting serum insulin/22.5

Abbreviations: HDL, high-density lipoprotein; IL-6, interleukin-6; TNF-α, tumor necrosis factor α.

*n* = number (male/female)

**Table 2:** Comparison of clinical and biochemical parameters after gender stratification

Clinical and biochemical parameters	Males		*P*-value	Females		*P*-value
	Met S (178)	Healthy group (165)		Met S (46)	Healthy group (35)	
Age in years	47.12 ± 8.23	46.27 ± 8.41	0.416	47.17 ± 7.08	45.11 ± 7.18	0.680
Systolic BP in mm of mercury	123.66 ± 15.14	114.38 ± 12.51	0.000^†^	131.14 ± 15.58	125.86 ± 30.91	<0.0001^†^
Diastolic BP in mm of mercury	80.07 ± 10.49	77.07 ± 7.62	0.000^†^	82.61 ± 10.30	80.55 ± 9.16	<0.0001^†^
Waist circumference in centimeters	101.65 ± 8.29	79.15 ± 8.54	0.000^†^	100.46 ± 11.35	78.62 ± 2.13	<0.0001^†^
Body mass index	28.46 ± 4.32	23.23 ± 3.61	0.000^†^	30.91 ± 5.73	24.99 ± 3.89	0.001^†^
HDL (mg/dl)	39.42 ± 8.38	39.12 ± 3.39	0.710	41.50 ± 12.3	41.60 ± 10.4	0.985
Triglycerides (mg/dl)	181 (132–262)	145 (119–164)	0.000*	215 (167–354)	150 (116–162)	<0.0001*
Cholesterol (mg/dl)	196 (175–240)	184 (164–200)	0.000*	214.5 (179–245)	177.5 (158–191)	0.003*
Serum uric acid in mg/dl	6.76 ± 1.53	5.30 ± 1.05	0.000^†^	6.13 ± 1.38	5.42 ± 1.06	0.172
Fasting glucose (mmol/l)	7.7 (4–10.7)	5.0 (4.4–5.3)	0.000*	9.74 ± 3.38	4.90 ± 1.35	<0.0001*
Fasting insulin (µg/ml)	29.81 (14.7–50.2)	8.85 (6.12–12.74)	0.000*	26.329 (18.4–46.3)	12.71 (10.60–16.70)	0.011*
HOMA-IR	10 (5.7–18.5)	1.88 (1.33–2.91)	0.000*	11 (7.8–17.5)	3.5 (2.12–4.80)	0.001*
TNF-α (pico g/ml)	20 (10–61)	9 (6–15)	0.000*	24 (9.7–64)	18 (10.5–74.5)	0.981
IL-6 (pico g/ml)	17 (6–47)	6 (4.3–16)	0.003*	12 (8–19.5)	14 (7–44)	0.425

Values given are Mean ± SD or Median (IQR) as related to the results obtained through the Shapiro–Wilk’s statistics. “*P”* value is generated by Student’s *t-* test^†^and Mann–Whitney *U-* test*. A *P* <0.05 is statistically significant.

HOMA-IR = Fasting serum glucose × Fasting serum insulin / 22.5 Abbreviations: HDL, high-density lipoprotein; IL-6, interleukin-6; TNF-α, tumor necrosis factor α. *n* = number (male/female)

### Correlation between serum cytokines and HOMA-IR and Met S related traits as per IDF guidelines

**Figure 1:**
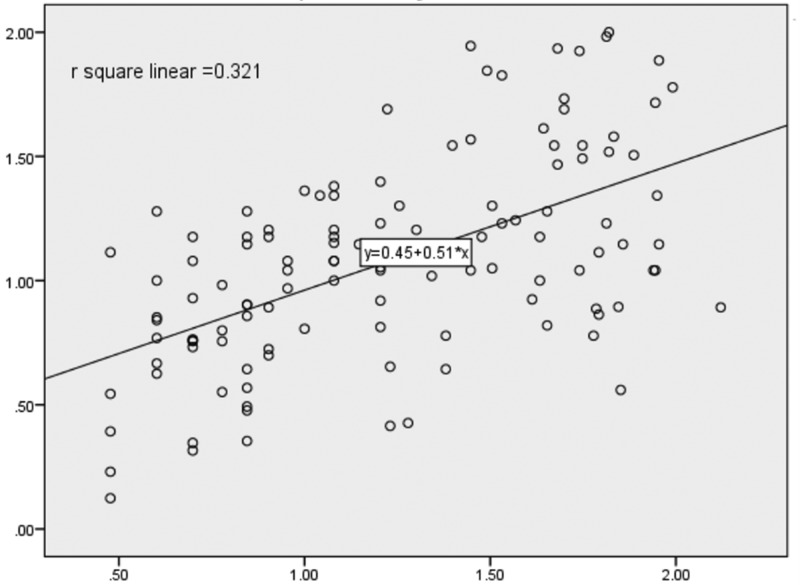
Scatter plot showing significant correlation (*P*=0.026*) between IL-6 and HOMA-IR in metabolic syndrome HOMA-IR and IL-6 were log transformed due to non-normal distribution.

**Figure 2:**
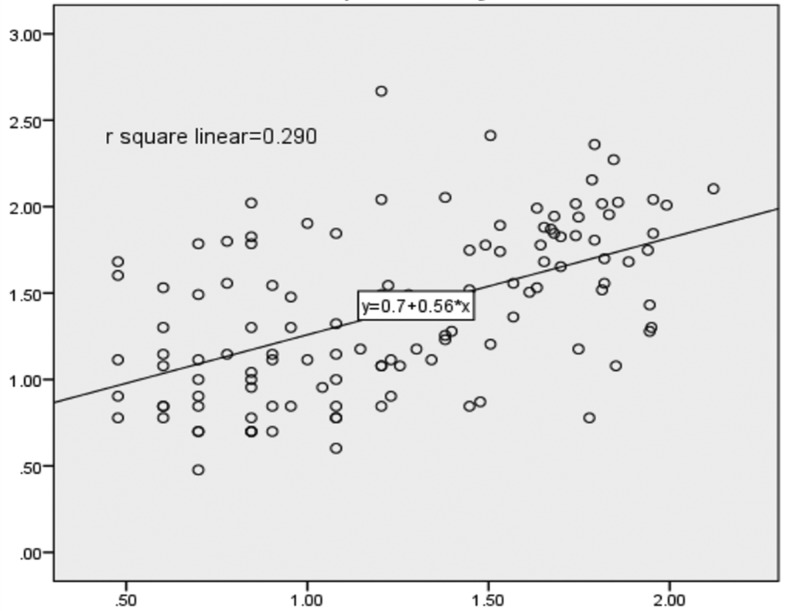
Scatter plot showing significant correlation (*P*=0.000*) between IL-6 and TNF-α in metabolic syndrome TNF-α and IL-6 were log transformed due to non-normal distribution.

Serum IL-6 and TNF-α showed significant positive correlation with HOMA-IR and with each other in Met S group while in control group only TNF-α showed significant correlation with HOMA-IR and no correlation was found between HOMA-IR and IL-6. When partial correlation was applied after controlling for the possible confounders such as age, sex, BP, waist circumference, serum lipid parameters and duration of the Met S, the correlation persisted between serum TNF-α and IL-6 (*P*=0.000) and serum IL-6 and HOMA-IR (*P*=0.000) but disappear between TNF-α and HOMA-IR in Met S group (Table 3; Figures 1 and 2).

**Table 3:** Correlation between the cytokines, HOMA-IR and Met S related traits according to the IDF guidelines

Spearman correlation	S.BP	D.BP	WC	HDL	Triglycerides	S.glucose	HOMA-IR	IL-6	TNF- α
	**Metabolic syndrome, *n*=200**								
IL- 6	0.076	0.186	0.155	−0.054	0.126	0.298	0.552	1.000	0.543
<0.0001*									
	0.410	0.044*	0.092	0.538	0.161	0.010*	0.026*		
TNF- α	0.043	0.101	0.107	−0.187	0.235	0.234	0.336	0.543	1.000
	0.641	0.275	0.234	0.041*	0.010*	0.001*	<0.0001*	<0.0001*	
	**Healthy group, *n*=100**								
IL- 6	0.019	0.094	0.169	0.251	0.213	0.433	0.161	1.000	0.396
	0.195	0.602	0.340	0.118	0.187	0.005*	0.328		0.002*
TNF- α	0.310	0.184	0.649	0.100	0.215	0.416	0.503	0.396	1.000
	0.079	0.306	<0.0001*	0.537	0.182	0.009*	0.001*	0.002*	

Spearman correlation was applied to see the relation between quantitative variables. Values of ‘*rho*’ given. A ‘*P*’ of <0.05 is statistically significant*.

Abbreviations: DBP, diastolic blood pressure; HOMA-IR, homeostatic model assessment for insulin resistance; Met S, metabolic syndrome; IDF, International Diabetes Federation; IL-6, interleukin-6; S. glucose, serum glucose; SBP, systolic blood pressure; TNF-α, tumor necrosis factor α; WC, waist circumference.

### Genotype frequency of TNF-α -308G>A rs1800629 in the study groups

In total study population frequency of homozygous dominant genotype GG was 47%, heterozygous GA was 42% and homozygous recessive AA was 11%. The frequency of G allele was 68% and A was 32%. Genotype frequency in the cases and the control was in Hardy–Weinberg equilibrium. On comparison of allelic, co-dominant (GG: GA: AA), dominant (GG: GA+AA) and recessive models (AA: GA+GG), there was no significant difference in frequency of genotypes between the Met S and the healthy groups (*P*>0.05) (Table 4).

**Table 4:** Comparison of TNF-α rs1800629 -308G>A in the study groups

Genotype	Metabolic syndrome, *n* (%age)	Healthy group, *n* (%age)	*P* value	OR and CI
**Co-dominant model**				
GG	100 (45)	102 (51)	0.337	3 × 2 OR not calculated
GA	101 (45)	76 (38)		
AA	23 (10)	22 (11)		
Total	224 (100)	200 (100)		
**Allelic frequency**				
G	301 (67)	280 (70)	0.357	0.87 (0.65–1.17)
A	147 (33)	120 (30)		
Total	448 (100)	400 (100)		
**Dominant model**				
GG	100 (45)	102 (51)	0.219	0.78 (0.53–1.15)
GA+AA	124 (55)	98 (49)		
Total	224 (100)	200 (100)		
**Recessive model**				
AA	23 (10)	22 (11)	0.876	1.05 (0.57–1.96)
GA+GG	201 (90)	178 (89)		
Total	224 (100)	200 (100)		

Chi square test was applied to calculate ‘*P*’ value, odds ratio (OR) and confidence interval (CI). A ‘*P*’ of <0.05 is statistically significant.

### Genotype frequency of IL-6 -174 G>C rs1800795 in the study groups

In total study population, frequency of homozygous dominant genotype (GG) was 45%, heterozygous (GC) was 45% and homozygous recessive (CC) was 10%. Genotype frequency in cases and control was in Hardy–Weinberg equilibrium. In the co-dominant model in Met S, frequency of genotype GG, GC and CC was 41%, 46% and 13%. In healthy group frequency of genotype GG, GC and CC was 50%, 44% and 6%. The frequency of CC genotype was significantly higher in Met S as compared with that of the healthy group (*P*=0.027). There was significant association of the recessive model (CC vs GC+GG) of IL-6 -174 G> C with Met S (odds ratio; 2.32 with CI; 1.15–4.70; *P*=0.016) (Table 5). Binary logistic regression analysis was applied taking the study groups (Met S and healthy group) as dependent variable to observe their association with the dominant and recessive genetic models of IL-6 -174 G>C variant. Adjusted *P* value was calculated after controlling the confounders such as age and sex. Recessive model of the IL-6 (CC vs GC+GG) was found to be significantly associated with Met S (*P*=0.020*, value of ‘Exp β’ 2.32 and CI: 1.14–4.67). No association of Met S was found with the dominant model of IL-6-174 G>C.

**Table 5:** Comparison of IL-6 rs1800795 -174 G>C in the study groups

Genotype	Metabolic syndrome, *n* (%age)	Healthy group, *n* (%age)	*P* value	OR & CI
**Co-dominant model**				
GG	92 (41)	100 (50)	0.027*	3 × 2 OR not calculated
GC	103(46)	88 (44)		
CC	29 (13)	12 (6)		
Total	224 (100)	200 (100)		
**Allelic frequency**				
G	287 (64)	288 (72)	0.013*	1.44 (1.07–1.93)
C	161 (36)	112 (28)		
Total	448(100)	400 (100)		
**Dominant model**				
GG	92 (42)	100 (50)	0.058	1.43 (0.97–2.11)
GC+CC	132 (58)	100 (50)		
Total	224 (100)	200 (100)		
**Recessive model**				
CC	29 (12)	12 (7)	0.016*	2.32 (1.15–4.70)
GC+GG	195 (88)	188 (93)		
Total	224 (100)	200 (100)		

Chi square test was applied to calculate the ‘*P*’ value, odds ratio (OR) and confidence interval (CI). A ‘*P*’ of <0.05 is statistically significant. After continuity correction adjusted *P* value were calculated by Chi-square test for the IL-6 co-dominant (*P*=0.024) and recessive model (*P*=0.025). The association remained significant.

### Gene expression analysis of IL-6, TNF-α and NFκB

**Figure 3:**
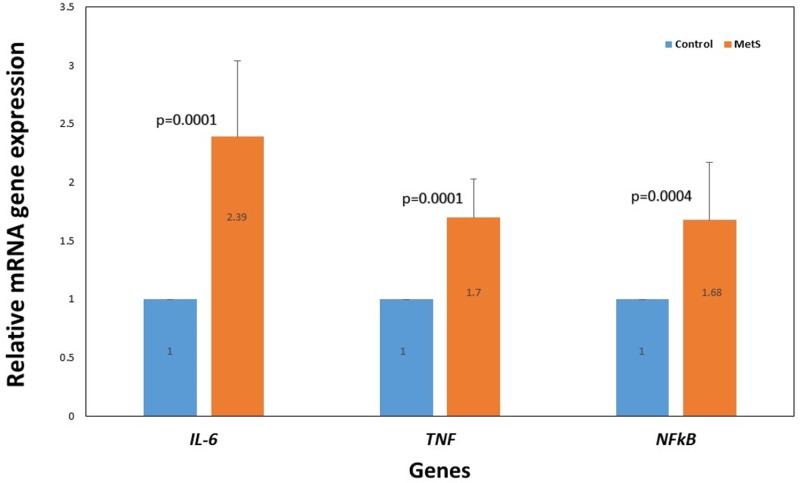
Graphical presentation of gene expression analysis of IL-6, TNF-α and NFκB in cases and controls

Gene expression analysis was done by using the software CFX96. Considering the expression of target genes in healthy controls as 1, IL-6, TNF-α and NFκB expression was significantly higher in cases than the controls (*P*<0.001 for IL-6 and TNF-α and *P* = 0.0004 for NFκB) (Figure 3).

### Comparison of serum IL-6 and TNF-α in different genotypes

On comparison of cytokine levels in different genotypes, TNF-α and IL-6 were significantly higher in AA and CC genotypes as compared with the more prevalent type GG (*P*=0.001 for TNF-α in cases and *P*=0.005 and 0.004 for IL-6 in cases and the controls). The *P*-value remained significant after Bonferroni adjustment for the multiple comparisons (for TNF-α, *P*=0.003; for IL-6, *P*=0.015 and 0.012 in cases and the controls) (Table 6).

**Table 6:** Comparison of serum cytokines in different genotypes in the study groups

Serum TNF-α in genotypes	GG (a)	GA (b)	AA (c)	*P*-value
Metabolic syndrome	13 (7–43)	30 (12–60)	68 (26–146)	0.060 (a:b)
				0.001* (a:c)
				0.015* (b:c)
Healthy group	14 (5.5–20.50)	8 (6–13.50)	18 (7–83)	0.675 (a:b)
				0.919 (a:c)
				0.835 (b:c)

Serum IL-6 in genotypes	GG (a)	GC (b)	CC (c)	*P*-value
Metabolic syndrome	16 (7–36)	16 (7–46)	70 (44–90)	0.516 (a:b)
				0.005* (a:c)
				0.010* (b:c)
Healthy group	7 (5–18.5)	5 (4–19)	20 (12–37)	0.668 (a:b)
				0.004* (a:c)
				0.002* (b:c)

Values given are Median (IQR) as the Shapiro–Wilk’s statistics stated data non-normal. ‘*P*’ value is generated by ‘Mann–Whitney *U*-test’ and a Bonferroni adjustment is applied. A *P*-value of ≤0.05 is statistically significant.

## Discussion

Visceral adiposity is the hallmark of IR. Altered innate immunity and infiltration of adipose tissue with macrophages is the basis of chronic low-grade inflammation leading to Met S [24]. TNF-α and IL-6 are inflammatory cytokines, released from peri-visceral fat and found to be associated with IR, endothelial dysfunction and atherogenesis [25]. In the present study, TNF-α and IL-6 were significantly higher in Met S (*P*<0.05) as compared with the controls and the two cytokines significantly correlated with HOMA- IR and each other. These results were supported by the earlier studies conducted on Ghanian and Iranian population reporting increased levels of TNF-α and IL-6 in T2DM and Met S; it was observed that cytokines acting in autocrine and paracrine fashion create a hostile environment of metabolic inflammation and insulin resistance [26,27]. IL-6 is a secondary cytokine released in response to the primary cytokine TNF-α; IL-6 induces hepatocytes to release CRP that highly correlates with atherosclerotic plaques, modulates innate immunity and contributes to IR [3,7,13]. In the present study, significant correlation was observed between serum IL-6 and TNF-α and this correlation remained significant even after controlling for the possible confounders, such as age, sex, BP and obesity markers. However, limited correlation was observed, between the IL-6 and the other Met S related traits like waist circumference, BP, serum HDL and triglycerides. This lack of association might be due to certain limitations of the present study. Majority of the cases were on treatment for diabetes mellitus, hypertension, and dyslipidemias; taking medications such as Glucophage, ACE inhibitors and statins. These drugs interfere with the release of cytokines and also with the insulin resistance, glycemic and metabolic control [28,29]. Duration of inflammation and Met S was also variable in the diseased group and this might also be responsible for this negative outcome. Despite of the above limitations of the study significant correlation between the cytokines and HOMA-IR persisted in Met S group after controlling for the possible confounders. In the present study, mRNA expression of IL-6, TNF-α and NFB was significantly higher in Met S as compared with the controls. These results are in concordance with the previous study, that also reported that the genes involved in the inflammatory and immune response pathway were significantly overexpressed in coronary artery disease and Met S cohort as compared with the one with rheumatoid arthritis [30]. In another study, decreased mRNA expression of TNF-α in peripheral white cells of obese subjects with Met S was observed after weight loss [31]. NFκB is one of the signal transduction pathways for the inflammatory cytokines. In normal circumstances NFκB is bound to its inhibitory protein IκB (inhibitors of κB) and is sequestered in the cell cytoplasm. During inflammation and insulin resistant states elevated levels of pro-inflammatory cytokines, insulin, glucose and free fatty acids all increase the activation of transcription factor NFκB by triggering the IKKB (IκB kinase) complex. Activation of IKKB results in phosphorylation and degradation of IκB protein. This exposes the nuclear localization sequence of NFκB, which directs the translocation of NFκB to the nucleus [32]. Here NFκB binds to the DNA response element of the target genes and triggers the production of inflammatory cytokines and chemokines [33]. NFκB-mediated cytokine production promotes the recruitment and activation of macrophages in the inflamed areas. The activated macrophages produce more cytokines and chemokines that further relocalize and activate macrophages to produce more cytokines [4]. Current results also depict that signaling pathway-NFκB status and the relevant cytokines expression was significantly raised in metabolic syndrome than the controls.

In the present study genetic variants of the promoter areas of TNF-α (-308 G>A rs1800629) and IL-6 (-174 G>C rs1800795) have been evaluated in the cases of Met S and the controls. The genotype GG of IL-6 was found to be the more prevalent type and CC the rarer one; the minor CC genotype showed significant association with the risk of Met S (*P*=0.016*, OR for CC vs GC+ GG; 2.32, CI; 1.15–4.70). These results were in concordance with those of the previous two studies in Pakistan reporting increased risk of CAD in subjects with CC genotype of IL-6 -174 G>C variant [15,34]. However another study, conducted on patients of rheumatic heart disease (RHD) in Pakistan reported, significant susceptibility to RHD in the GG mutants of IL-6-174G>C [35]. The results based upon the previous studies to determine the risk allele of IL-6 -174G>C polymorphism for the various adverse outcomes are conflicting. The major GG genotype in the promoter area of IL-6 -174 G>C was found to be associated with hypertension and increased plasminogen activator inhibitor-1 in Taiwanese. The possible relation was mediated through the inflammatory mechanisms resulting in the development and instability of the atherosclerotic plaque [36]. The GG variant of the promoter polymorphism of IL-6 was also reported to be the significant predictor of early death after acute coronary syndrome in elderly males [37]. On genotype analysis, the major allele G of IL-6 -174G>C was reported to be a risk factor for type 2 diabetes mellitus in Indian population [38]. However in another study, CC genotype was found to be associated with carotid atherosclerotic plaques in Portuguese [39]. Results of the previously published studies were also variable regarding the association of the TNF-α -308 G>A genotype with the various diseased states. Linkage of the TNF-α AA genotype has been reported with the increased TNF-α expression, rheumatic heart disease, myocardial infarction and ischemic stroke [9,12,40]. On the contrary, a case–control study on North Indians revealed no association between TNF genotypes and T2DM [41]. Another study by Philips et al. reported an association of major (G) allele of TNF-α -308 G>A with Met S and related traits {*P*=0.002 OR 1.35 (CI [37]; 1.02–1.7)} [42]. In the present work, there was no significant association of TNF-α -308 G>A genotype with Met S in Pakistanis. On comparison of cytokines in different genotypes, levels of cytokines varied with their respective genetic variations. TNF-α and IL-6 levels were significantly higher in the minor genotypes; AA and CC of TNF-α -308 G>A and IL-6 -174 G>C as compared with the wild-type (*P*=0.001). Met S also labeled as inflammatory syndrome might be responsible for the increased cytokine levels in the diseased group but IL-6 was significantly raised in the controls as well. IL-6 also showed significant correlation with IR and TNF-α. These findings were consistent with the similar observations on the patients of the premature CAD from Rawalpindi, Pakistan [34]. Genetic variants present in the promoter or regulatory areas of the cytokines can act as potential immune regulators by controlling the expression of cytokines. There can be person to person variations in cytokine levels depending upon the genetic coding-resulting in diverse immune response [43].

## Conclusion

In the present study, there was significant association of CC genotype of IL-6 -174 G>C with the Met S. On comparison of cytokine levels in different genetic models serum ‘IL-6’ levels were significantly higher in the CC genotype of IL-6 -174 G>C as compared with the others (*P*=0.001) in cases and the controls. Identification of the cytokine gene variants and their association with insulin resistance traits might help us in identifying the high-risk individuals. Immune-mediated therapies targeting cytokines or resident macrophages of adipose tissue in obese individuals might be helpful as therapeutic options to reverse insulin resistance and prevent organ damage in type 2 diabetic obese patients by limiting the pro-inflammatory environment.

## Abbreviations

BP: blood pressure
CAD: coronary artery disease
HDL-c: high-density lipoprotein cholesterol
HOMA-IR: homeostatic model assessment for insulin resistance
IL-6: interleukin-6
IR: insulin resistance
IRS-1: insulin receptor substrate1
Met S: metabolic syndrome
RFLP: restriction fragment length polymorphism
RHD: rheumatic heart disease
TGS: serum triglycerides
SNP: single nucleotide polymorphism
T2DM: type 2 diabetes mellitus
TNF-α: tumor necrosis factor α
